# Diffuse non-midline glioma with *H3F3A* K27M mutation: a prognostic and treatment dilemma

**DOI:** 10.1186/s40478-017-0440-x

**Published:** 2017-05-15

**Authors:** Giselle López, Nancy Ann Oberheim Bush, Mitchel S. Berger, Arie Perry, David A. Solomon

**Affiliations:** 1 Division of Neuropathology, Department of Pathology, University of California, San Francisco, 513 Parnassus Ave, Health Sciences West 451, Box 0102, San Francisco, CA 94143 CA USA; 2 Division of Neuro-Oncology, Department of Neurological Surgery, University of California, San Francisco, CA USA; 3 Department of Neurological Surgery, University of California, San Francisco, CA USA; 4 Clinical Cancer Genomics Laboratory, Department of Pathology, University of California, San Francisco, CA USA

Recent studies have identified that K27M mutation in either the *H3F3A* or *HIST1H3B* genes, which encode the histone H3 variants H3.3 and H3.1, define the majority of diffuse gliomas arising in midline structures including the thalamus, brainstem, and spinal cord in both children and adults [8, 10]. These “diffuse midline gliomas, H3 K27M-mutant” are associated with poor prognosis regardless of histologic grade and were thus designated as a grade IV entity in the 2016 WHO Classification [3]. Here we illustrate that H3 K27M mutation can occur in cortically-based diffuse gliomas not arising in midline structures and discuss the uncertainties regarding grading and prognostic classification for such tumors.

A 20-year-old woman presented with several years of seizures characterized by right-sided dysesthesia that were increasing in frequency. Magnetic resonance imaging demonstrated a 2.2 cm expansile mass centered in the left insular cortex with patchy contrast enhancement (Fig. 1a, Additional file 1: Figure S1). Craniotomy and gross total resection of the mass was performed. H&E stained sections demonstrated an infiltrative glial neoplasm composed of cells with markedly pleomorphic nuclei, coarse granular chromatin, and scant cytoplasm (Fig. 1b, Additional file 1: Figure S2). Perineuronal satellitosis and perivascular accumulation of tumor cells was prominent. The mitotic index was low, averaging less than 1 mitosis per 10 high power fields. Neither microvascular proliferation nor necrosis was identified, nor were there any Rosenthal fibers, eosinophilic granular bodies, or dysmorphic ganglion cells. The Ki-67 labeling index was estimated at 2%. The tumor cells were negative for IDH1 R132H mutant protein and had intact ATRX expression. A preliminary diagnosis of “diffuse astrocytic neoplasm with WHO grade II histologic features” was rendered. Targeted next-generation sequencing was performed on the UCSF500 Cancer Panel as previously described to clarify the molecular subtype [2]. This identified an *H3F3A* p.K27M mutation, an *ATRX* p.2194delQ mutation, and a novel *BRAF* gene fusion predicted to result in an in-frame fusion protein with the N-terminal portion composed of exons 1–11 of *EPB41L2* and the C-terminal portion composed of exons 10–18 of *BRAF*, which encode the serine/threonine kinase domain (Fig. 1d, Additional file 1: Figure S3). The *ATRX* mutation localizes within the C-terminal helicase domain of the encoded protein where the majority of the non-truncating missense mutations in this gene cluster and was thus considered likely to be pathogenic. Chromosomal copy number changes in the tumor were limited to gain of 1q and loss of 22q. No alterations involving *IDH1*, *IDH2*, *ACVR1*, *PPM1D*, *BCOR*, *EGFR*, *PTEN*, *NF1*, *SETD2*, or *TP53* were identified. Subsequent immunostaining of the tumor for H3 K27M mutant protein confirmed the presence of nuclear expression, combined with the expected loss of histone H3 lysine 27 trimethylation (Fig. 1c, Additional file 1: Figure S2). Expression of H3 K27M mutant protein was observed in all of the tumor nuclei, suggesting that it was likely an early or initiating event in this patient’s tumor. As only a couple prior examples of such cortically-based diffuse gliomas with H3 K27M mutation have been reported [7, 9], the prognostic significance of this combination of histologic and genetic features is uncertain at present, as is optimal therapy. Adjuvant radiation and chemotherapy with temozolomide were recommended, but the patient opted to seek consultation from other academic medical centers.

**Fig. 1:**
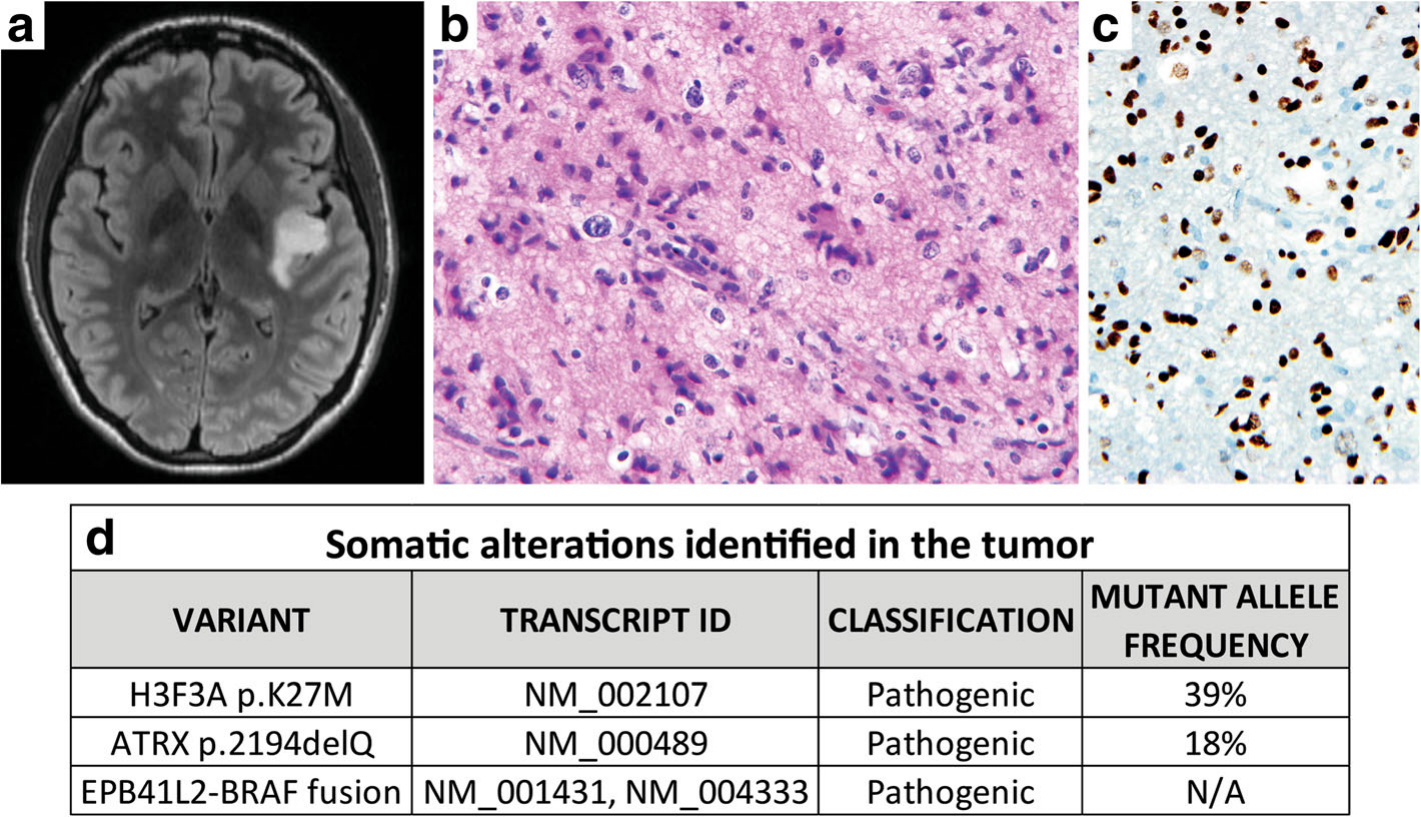
Radiographic, histologic, and genetic features of a cortically-based diffuse non-midline glioma with histone H3 K27M mutation. **a**, Axial T2 FLAIR magnetic resonance image. **b**, H&E stained section of the tumor. **c**, Immunostain for histone H3 K27M mutant protein. **d**, Genetic alterations identified in the tumor by next-generation sequencing

While common in diffuse midline gliomas, H3 K27M mutation appears to be a rare genetic alteration in diffuse gliomas arising peripherally in the cerebral hemispheres. Initial reports documented that diffuse midline gliomas with H3 K27M mutation are associated with a uniformly poor prognosis; however, these tumors are centered in critical midline structures such as the brainstem and spinal cord, thereby preventing surgical resection in most cases. It is unclear to what extent the poor prognosis of these tumors is due to the inability of resection versus the biologic behavior caused by the H3 K27M mutation. As some cortically-based diffuse gliomas can be gross totally resected (as was the case in this patient), the prognosis and need for aggressive adjuvant therapy in this setting is therefore uncertain. Also of note is a recent study suggesting that diffuse thalamic gliomas in adults harboring H3 K27M mutation are not associated with a uniformly poor prognosis [1], as well as a few reports of circumscribed low-grade glial neoplasms centered in midline structures that harbor H3 K27M mutation. These cases histologically resembled ganglioglioma or pilocytic astrocytoma and were associated with more indolent disease course than typical diffuse midline gliomas [4–6]. Together with these reports, this patient demonstrates that H3 K27M mutation is not limited to diffuse midline gliomas and that more studies are need to define the prognosis and optimal treatment for the growing spectrum of both midline and non-midline tumors that harbor this critical oncogenic mutation. We suggest that immunostaining for H3 K27M mutant protein be considered in all IDH-wildtype diffuse gliomas in young patients, not just those centered in midline structures. However, we emphasize that only those diffuse gliomas centered in midline structures harboring H3 K27M mutation fulfill the diagnostic criteria for the entity “diffuse midline glioma, H3 K27M-mutant” classified as grade IV per the 2016 WHO Classification. As the prognosis for those circumscribed gliomas or diffuse non-midline gliomas with H3 K27M mutation remains uncertain at present, these tumors should not be designated as WHO grade IV.

## Additional file

Additional file 1: Figure S1. Imaging features of the diffuse non-midline glioma, H3 K27M-mutant. **Figure S2.** Histologic features of the diffuse non-midline glioma with histone H3 K27M mutation. **Figure S3.** Genetic features of the diffuse non-midline glioma with histone H3 K27M mutation. (PDF 6168 kb)
