# Supplementary material for: Diffuse non-midline glioma with H3F3A K27M mutation: a prognostic and treatment dilemma
Source: Acta Neuropathol Commun. 2017 May 15;5:38. doi: 10.1186/s40478-017-0440-x (PMC5433088; doi:10.1186/s40478-017-0440-x)
Supplement: Additional file 1: Figure S1. — Imaging features of the diffuse non-midline glioma, H3 K27M-mutant. Figure S2. Histologic features of the diffuse non-midline glioma with histone H3 K27M mutation. Figure S3. Genetic features of the diffuse non-midline glioma with histone H3 K27M mutation. (PDF 6168 kb) [file 40478_2017_440_MOESM1_ESM.pdf]

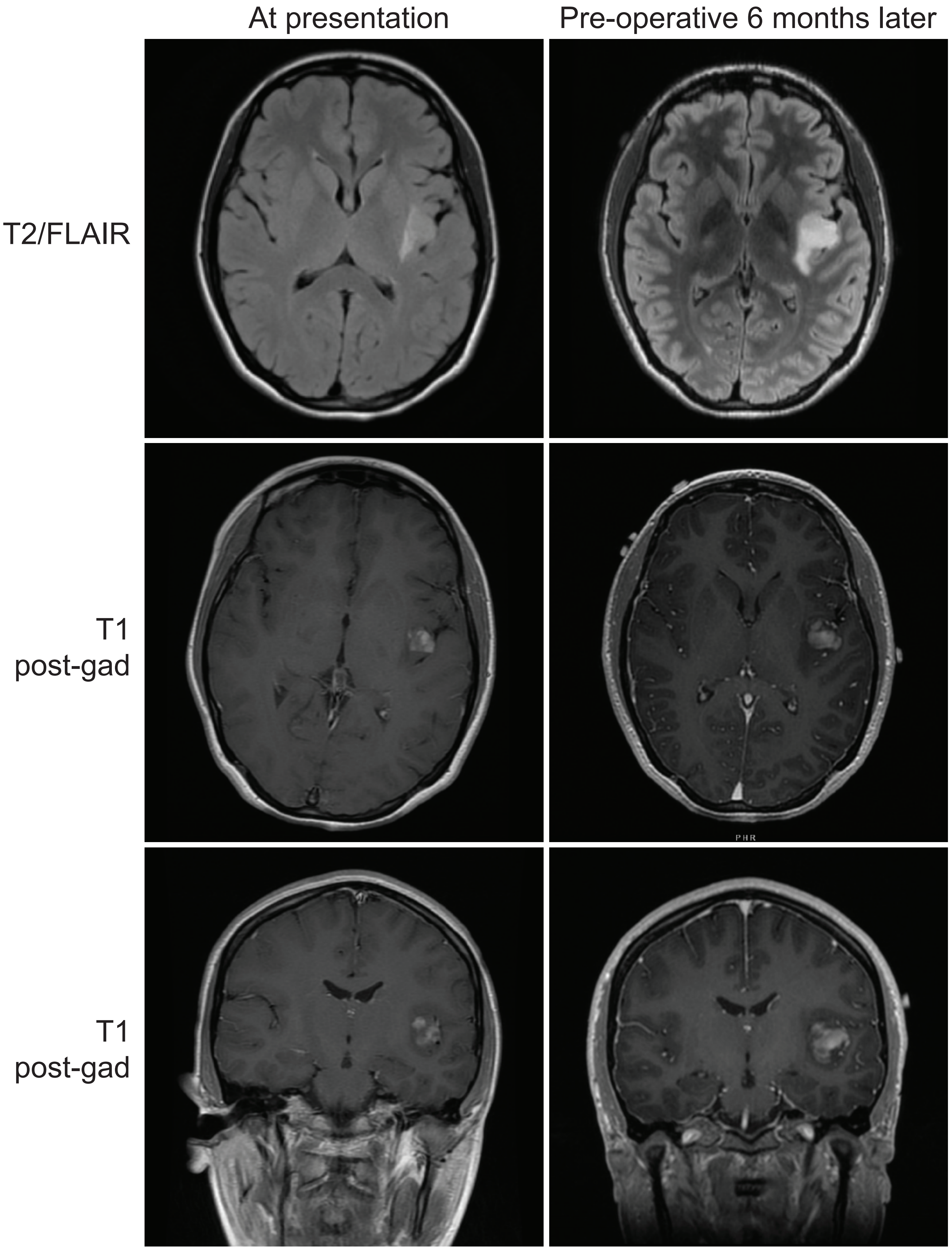

Supplemental Figure 1. Imaging features of the diffuse non-midline glioma, H3 K27M-mutant.

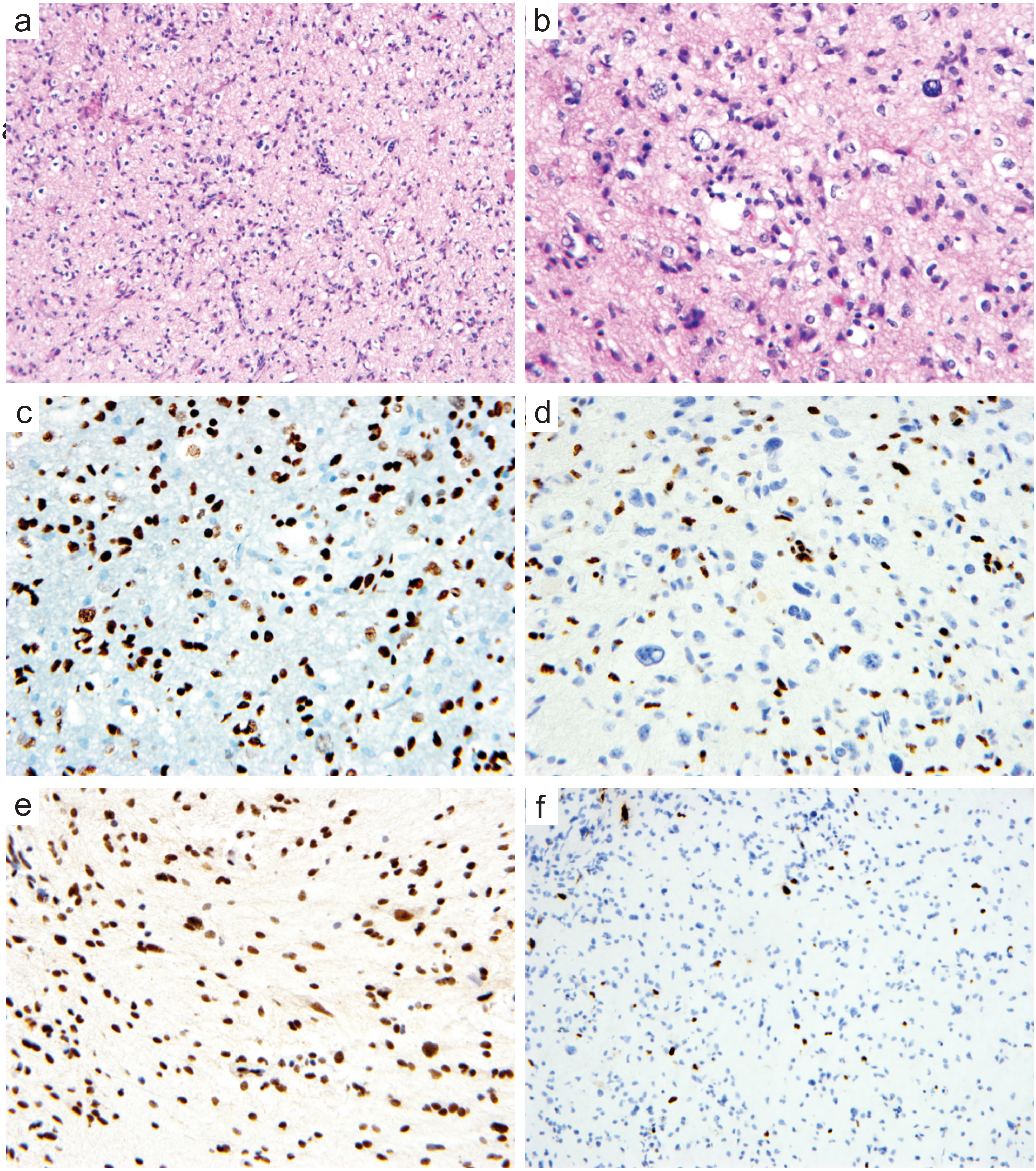

Supplemental Figure 2. Histologic features of the diffuse non-midline glioma with histone H3 K27M mutation. **a,b** Hematoxylin and eosin stains. **c** Immunostain for histone H3 K27M mutant protein. **d** Immunostain for histone H3 lysine 27 trimethylation. **e** Immunostain for ATRX protein. **f** Ki67 immunostaining.

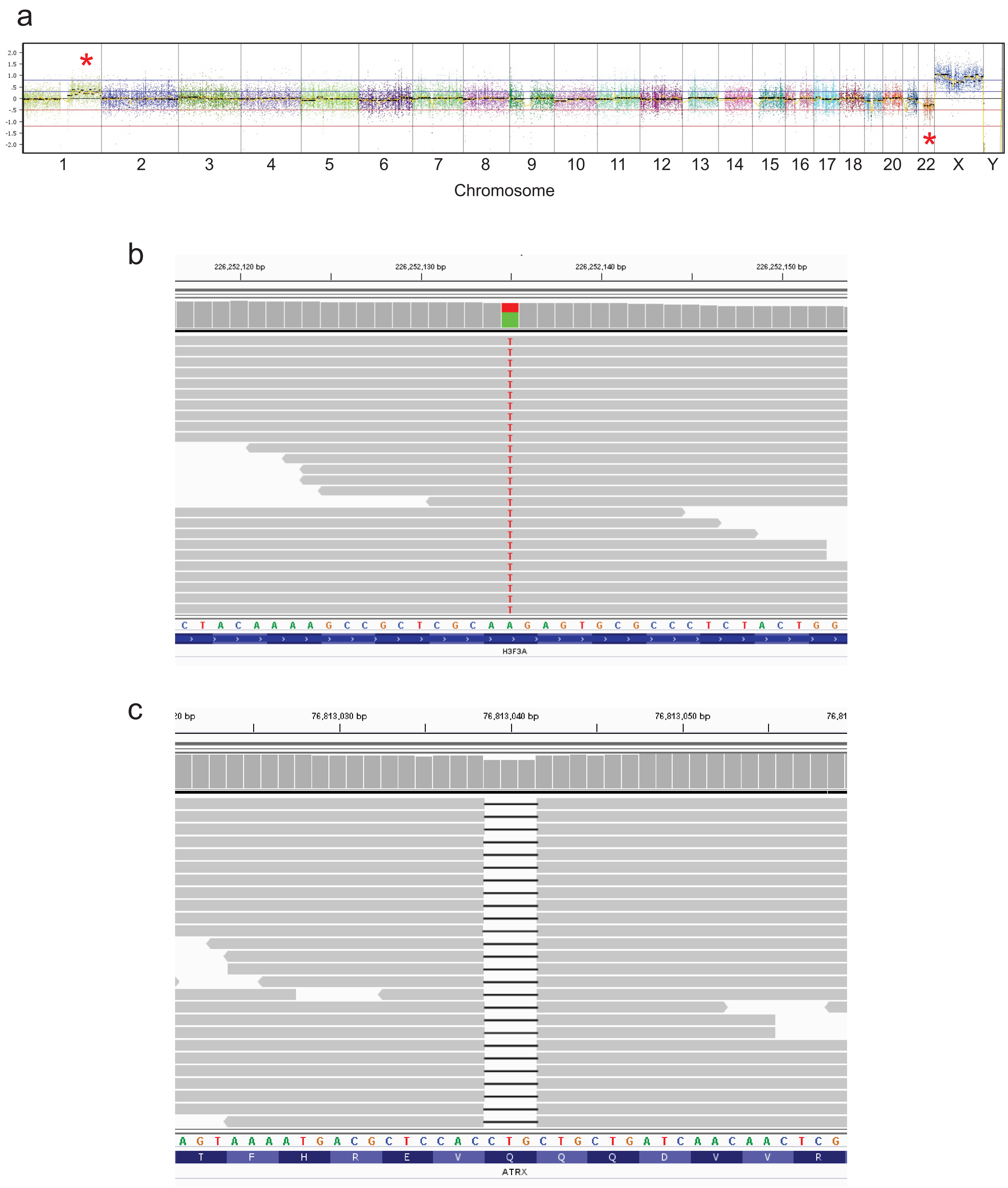

a
2.0
1.5
1.0
.5
0
-.5
-1.0
-1.5
-2.0
*
*
1 2 3 4 5 6 7 8 9 10 11 12 13 14 15 16 17 18 20 22 X Y
Chromosome
b
226,252,120 bp
226,252,130 bp
226,252,140 bp
226,252,150 bp
C T A C A A A A G C C G C T C G C A A G A G T G C G C C C T C T A C T G G
H3F3A
c
76,813,030 bp
76,813,040 bp
76,813,050 bp
A G T A A A A T G A C G C T C C A C C T G C T G C T G A T C A A C A A C T C G
T F H R E V Q Q Q D V V R
ATRX

d

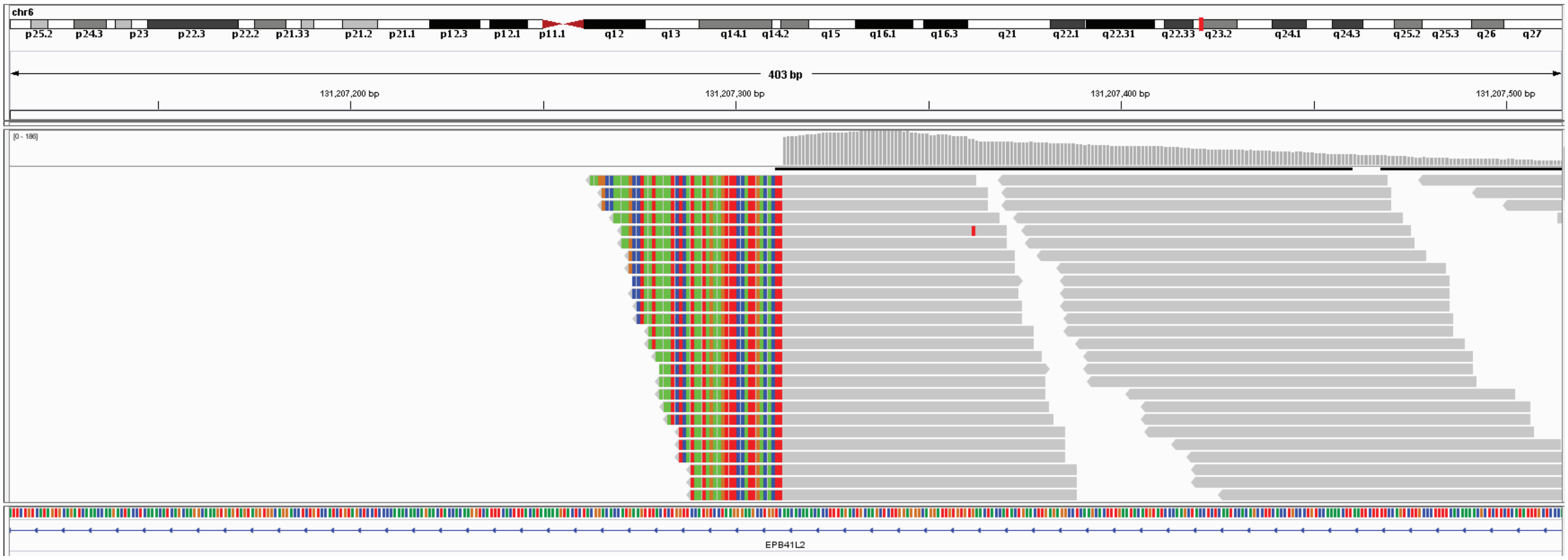

e

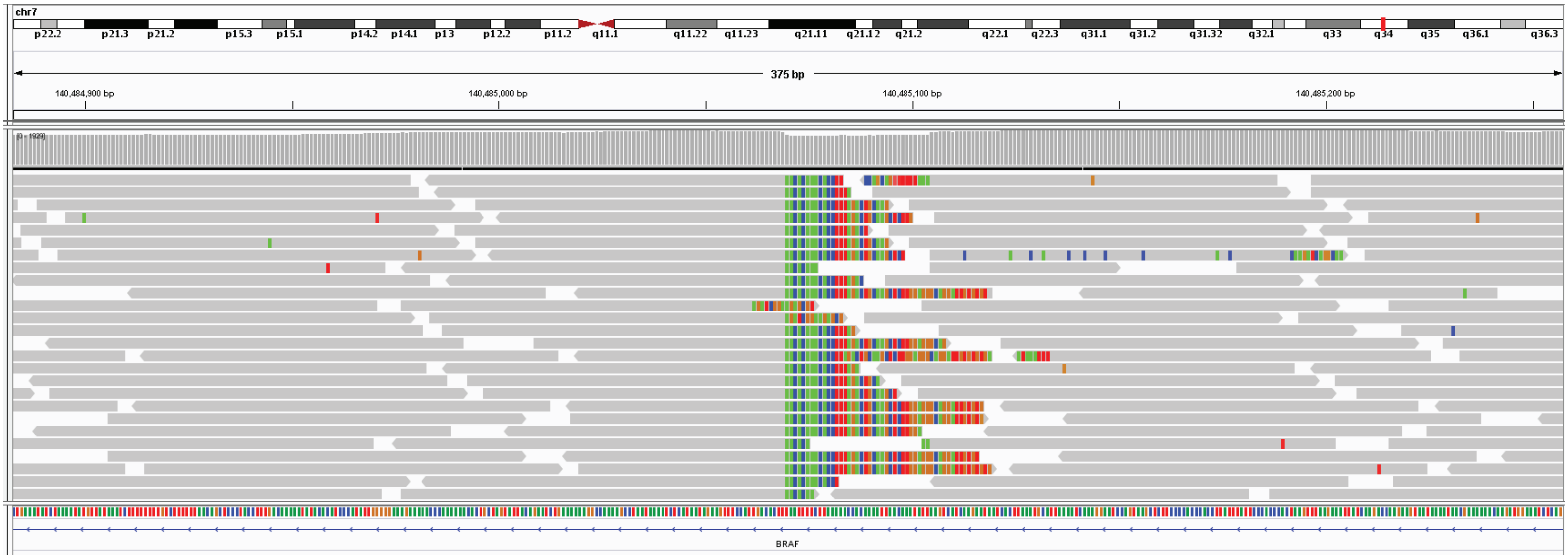

Supplemental Figure 3. Genetic features of the diffuse non-midline glioma with histone H3 K27M mutation. **a** Genome-wide copy number plot of the tumor showing gain of chromosome 1q and loss of 22q. **b** Sequencing reads over the *H3F3A* p.K27M mutation. **c** Sequencing reads over the *ATRX* p.2194delQ mutation. **d** Sequencing reads over the *EPB41L2-BRAF* fusion junction within intron 11-12 of *EPB41L2*. **e** Sequencing reads over the *EPB41L2-BRAF* fusion junction within intron 9-10 of *BRAF*.
